# Observations on the Physiological Anatomy of the Infant’s Stomach Noted in the Course of the Use of a Balloon-Duodenal Catheter

**Published:** 1914-07

**Authors:** 


**Observations on the Physiological Anatomy of the Infant's Stomach Noted in the Course of the Use of a Balloon-Duodenal Catheter.** Hess, in the American Journal of Diseases of Children, June, 1914, describes a new instrument which he calls the duodenal balloon catheter, by means of which he proves that the gastric canal (Magenstrasse of Waldeyer) of the infant is much more nearly a vertical street, or path, than a horizontal one, and illustrates this by Röntgen pictures, though the stomach of the infant lies horizontally. With this instrument he has also been able to produce cardiospasm, which he thinks may be due to increased pressure within the gastric cavity.—C. G. L-W.

